# Robot Evolution: Ethical Concerns

**DOI:** 10.3389/frobt.2021.744590

**Published:** 2021-11-03

**Authors:** Ágoston E. Eiben, Jacintha Ellers, Gerben Meynen, Sven Nyholm

**Affiliations:** ^1^ Department of Computer Science and Ecological Science, Vrije Universiteit Amsterdam, Amsterdam, Netherlands; ^2^ Department of Electronic Engineering, University of York, York, United Kingdom; ^3^ Department of Ecological Science, Vrije Universiteit Amsterdam, Amsterdam, Netherlands; ^4^ Department of Philosophy, Vrije Universiteit Amsterdam, Amsterdam, Netherlands; ^5^ Department of Law, Utrecht University, Utrecht, Netherlands; ^6^ Department of Philosophy and Religious Studies, Utrecht University, Utrecht, Netherlands

**Keywords:** evolutionary robotics, evolutionary design, ethics, meaningful human control, responsibility gaps, real-world robot evolution, morphological robot evolution

## Abstract

Rapid developments in evolutionary computation, robotics, 3D-printing, and material science are enabling advanced systems of robots that can autonomously reproduce and evolve. The emerging technology of robot evolution challenges existing AI ethics because the inherent adaptivity, stochasticity, and complexity of evolutionary systems severely weaken human control and induce new types of hazards. In this paper we address the question how robot evolution can be responsibly controlled to avoid safety risks. We discuss risks related to robot multiplication, maladaptation, and domination and suggest solutions for meaningful human control. Such concerns may seem far-fetched now, however, we posit that awareness must be created before the technology becomes mature.

## Introduction

Surprisingly, the idea of robot evolution is one hundred years old. The famous play by Karel Čapek that coined the word “robot” was published in 1920 (Čapek 1920). Towards the end of the play the robots are at the verge of extinction and one of the humans, Alquist, advises them: “*If you desire to live, you must breed like animals.”* In 1920 this was a fantastic idea–as in: impossible. In today’s world with rapidly proliferating artificial intelligence and robotics it is still a fantastic idea, but not impossible anymore.

Towards the end of the twentieth century the principles of biological evolution were transported to the realm of technology and implemented in computer simulations. This brought on the field of Evolutionary Computing, and evolutionary algorithms proved capable of delivering high quality solutions to hard problems in a variety of scientific and technical domains, offering several advantages over traditional optimization and design methods (Ashlock 2006; de Jong, 2006; Eiben and Smith 2003). Evolutionary algorithms have also been applied to developing the morphology (the hardware “body”) and controller (the software “brain”) of autonomous robots, which resulted in a new field called Evolutionary Robotics (Nolfi and Floreano 2000; Bongard 2011; Vargas et al., 2014; Doncieux et al., 2015).

**FIGURE 1:**
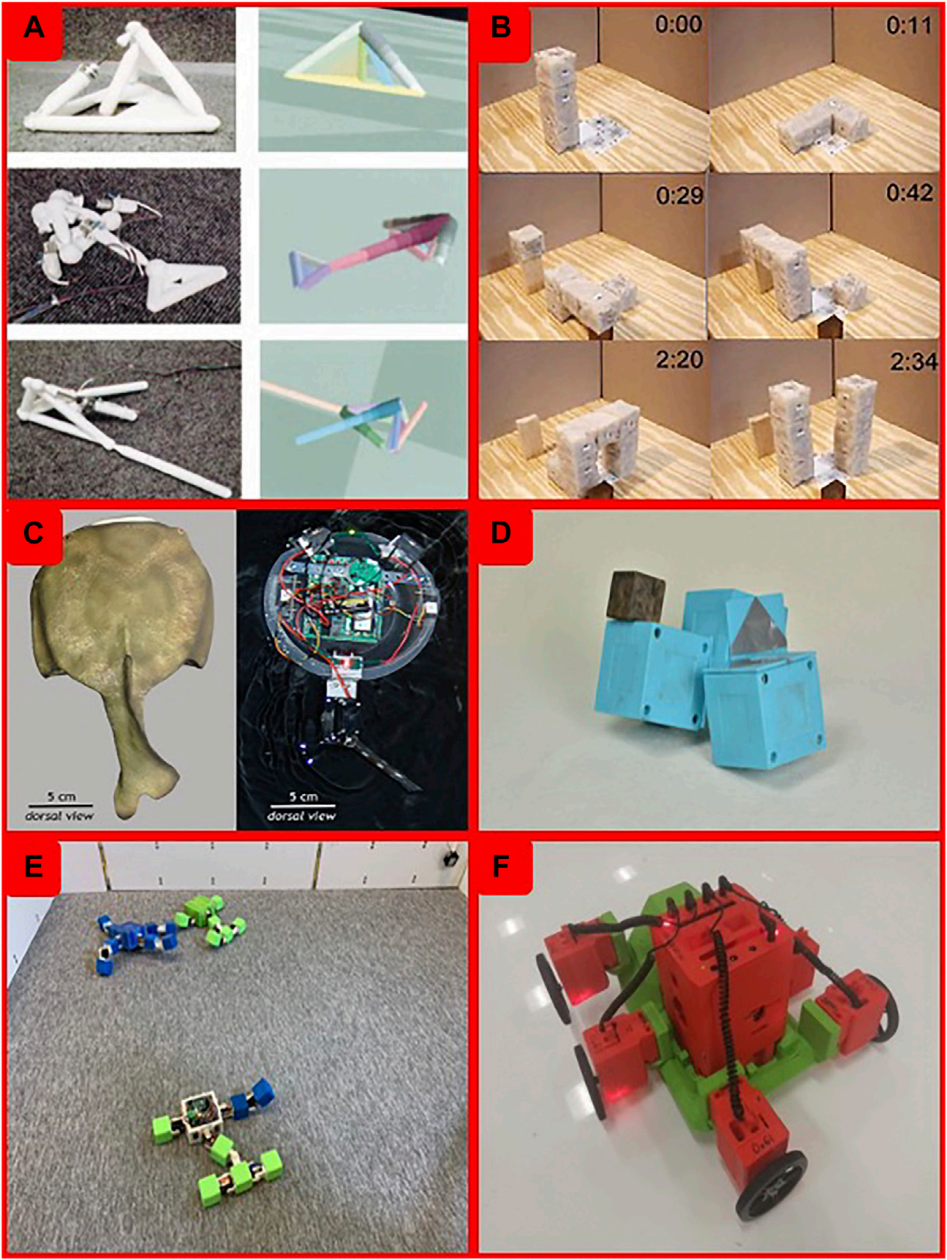
Some of the landmarks of the history of robot evolution. We show examples of systems that demonstrated robot reproduction or evolution incarnated in the real world. **(A)** 2000: The GOLEM project (10) co-evolved robot bodies and controllers in simulation and fabricated the evolved robot afterwards. **(B)** 2005: A physical system based on Molecubes, demonstrated non-adaptive robots able to construct a replica of themselves (*12*). **(C)** 2012: Tadro robots (*13*) were used to verify a hypothesis about the evolution of Cambrian vertebrates. Consecutive generations were constructed and evaluated in real hardware. **(D)** 2015: Semi-automated construction of genetically encoded modular robots (15). Consecutive generations were constructed and evaluated in real hardware. **(E)** 2016: The Robot Baby Project (*17*) demonstrated the reproduction of genetically encoded robots. Robots co-existed in the same environment; the offspring was added there after “birth.” **(F)** 2019: The Autonomous Robot Evolution Project (*18*) features hands-free construction of genetically encoded robots. The robots have sensors and can co-exist in the same environment. The robots shown in **(1A,C–E)** had no sensors. The robots shown in **(1C,D)** were constructed and evaluated one by one; the physical population consisted of one single robot at any time. The robots in **(1B)** are actually not evolvable, as there was no genetic encoding and the replica was an identical copy.

Up till now, work on evolutionary robotics has mostly been performed in computer simulations, safely confined to a virtual world inside a computer [e.g (Bongard 2011)]. Occasionally, the best robots in the final generation have been constructed and materialized in the real world (Lipson and Pollack 2000; Kriegman et al., 2020), but even in these cases the evolutionary process itself took place in simulation. Some studies have demonstrated self-reproducing physical machines, but the resulting system was not evolutionary because there was no inheritance and reproduction created identical clones without variation (Zykov et al., 2005). Research about robots that reproduce and evolve in the real world has been rare because of technical limitations in the (re)production of arbitrary robot shapes (Long 2012). In Figure 1 we exhibit some of the landmarks of the history of robot evolution.

**FIGURE 2:**
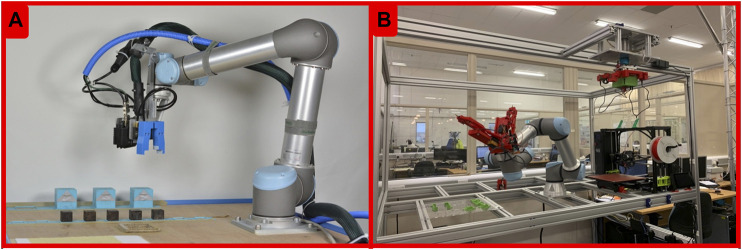
Examples of robot reproduction facilities. Photos of two (semi) automated robot reproduction facilities. **(A)**: the system used in Cambridge (15). **(B)**: the one used in Bristol (18).

However, this situation is changing rapidly and after the first major transition from “wetware” to software in the 20th century, evolution is at the verge of a second one, this time from software to hardware (Eiben and Smith 2015). Recent advances in and integration of evolutionary computation, robotics, 3D-printing, and automated assembly are enabling systems of physical robots that can autonomously reproduce and evolve (Brodbeck et al., 2015; Jelisavcic et al., 2017; Vujovic et al., 2017; Hale et al., 2019; Howard et al., 2019; Ellery 2020). The key concepts behind robots evolving in the real world are explained in Box 1, while Box 2 illustrates how the most challenging step of the process, robot reproduction, can be implemented. Two examples of existing robot reproduction facilities are shown in Figure 2. Such autonomous evolutionary systems incarnated in hardware offer advantages for applications as well as for fundamental research.^1^

BOX 1 **Robots evolving in the real world**

To make robots evolvable selection and reproduction need to be implemented. Selection of “robot parents” can be done by evaluating the robot’s behavior and allocating higher reproduction probabilities to robots that work well. For reproduction two facets of a robot should be distinguished, the ***phenotype*** that is the physical robot itself and the ***genotype*** that is the specification sheet, the robotic equivalent of DNA that describes and encodes the phenotype. Reproduction can then be defined through two principal steps. The first step is to create a new genotype that encodes the offspring. This step generates genetic variation either by a recombination operator that stochastically mixes the genotypes of two parents (sexual reproduction) or by a mutation operator that causes random changes in the genotype of one single parent (asexual reproduction). This step is a fully digital operation that can use existing methods from traditional Evolutionary Computation. The second step is the execution of the genotype-phenotype mapping, that is, the construction of the physical robot offspring as specified by the newly produced genotype. A crucial technical challenge in robot evolution lies in the second step, the production of offspring.

BOX 2 **Robot (re)production**

A robotic genotype obtained by mutating the genotype of one robot or recombining the genotypes of two parent robots encodes a new robot, the offspring. This offspring could be constructed by feeding the genotype to a 3D printer that makes a robot as specified by this genotype. However, currently there are no 3D printers that can produce a fully functional robot including a CPU, battery, sensors, and actuators. Arguably, this problem is temporary, and rapid prototyping of such components will be possible in the (near) future. A practicable alternative for now is to combine 3D printing, prefabricated functional components stored in a repository (e.g., CPUs, batteries, sensors, and actuators), and automated assembly. In such a system, the genotype specifies a number of 3D printable body parts with various shapes and sizes, the types, numbers and geometrical positions of the prefabricated body parts and the properties of an adequate software “brain” to control the given body. The production of a new robot can be done by industrial robot arms that retrieve the 3D printed body parts from the printers, collect the necessary prefabricated components from the storage, and assemble them into a working robot. After that, the software can be downloaded and installed on the CPU and the new robot can be activated.

**FIGURE 3:**
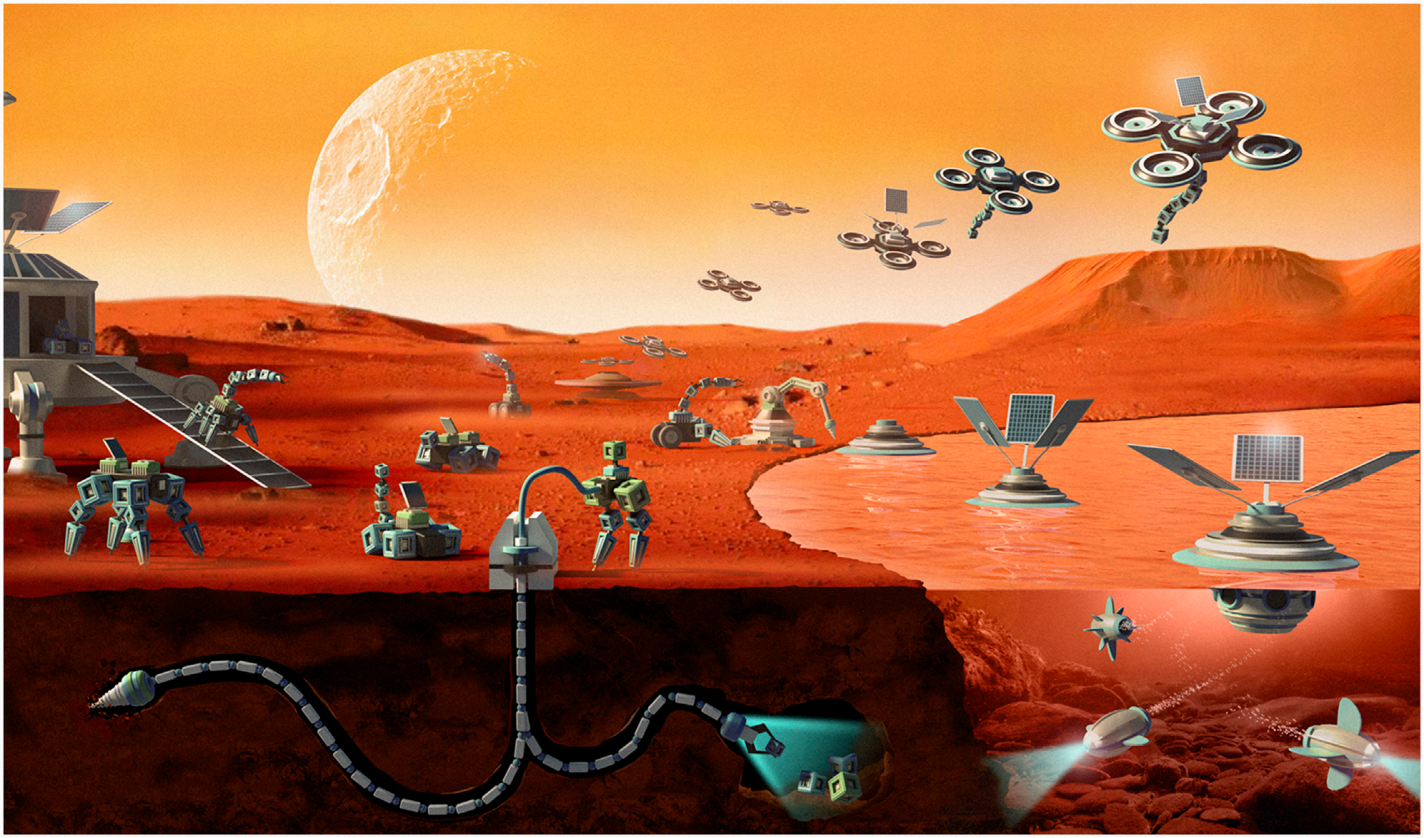
Artist impression of evolving robots in space.

For practitioners, evolution serves as an approach to adjust optimal robot designs on-the-fly in dangerous or inaccessible places [19], such as mines, nuclear power plants, or even extraterrestrial locations (see Figure 3). Additionally, evolving robots can be seen as hardware models of evolutionary systems [13]. Thus, they can be used as a new type of research instrument for testing hypotheses about biological processes (Nolfi and Floreano 2000) and deliver deeper understanding of universal evolutionary principles (Floreano and Keller 2010; Waibel et al., 2011). Autonomous robot evolution can thus be a game changer compared to evolutionary systems implemented in the digital realm (Eiben et al., 2012).

A key insight of this paper is that the science and technology of robot evolution are elevating the known concerns regarding AI and robotics to a new level by the phenomenon we call *second order engineering* or *second order design*. First order system engineering is the current practice where AI and robots are developed and engineered directly by humans. Evolutionary robot technology radically changes this picture because it introduces a new layer: instead of directly constructing a robotic system for a certain application, humans are constructing an evolutionary system that will construct a robotic system. Ethical, moral and safety concerns should therefore be converted into design principles and methodological guidelines for humans. The fundamental challenge here is the inherent stochasticity and complexity of an evolutionary system and the weakened influence of humans on the end product. This implies that all issues of the current discourse on AI and robot ethics remain valid [see, e.g. (Torresen 2018)], but that we also get new ones.

The new ethical challenges related to robot evolution are rooted in the inherent inefficiency and unpredictability of the evolutionary process. Evolution proceeds through the generation of heritable variation (recombination and mutation) in combination with selection that favors more successful forms at the cost of large numbers of failures (Futuyma 2013). Evolving robots in hardware through automated (re)production may therefore bring about a high number of arbitrary robot forms, which increases the chance of unintentionally creating robots with harmful behaviors. Moreover, key evolutionary changes often take place in the form of large unpredictable innovations that arise from rearrangements of existing characteristics for new functions (True and Carroll 2002). Such emergent evolution is highly unpredictable in both direction and magnitude, increasing the likelihood that evolving robots will have unexpected capacities.

Whenever there is a technology that is not directly under human control–technologies without a “steering wheel”–and whenever the process is unpredictable, questions about risks and responsibilities arise (Sparrow 2007; Hansson 2017; Nihlen Fahlquist 2017; Santoni de Sio and van den Hoven 2018; Nyholm 2020). Do the benefits of the new technology outweigh its possible adverse effects? If there are adverse effects, how can we minimize and control these? And, importantly, if things spin out of control, who is responsible? Answering these questions not only requires solutions from the field of robot evolution itself, but also raises ethical issues about the measures we should take to prevent harm. One could argue that such concerns are far-fetched. However, we posit that these issues must be addressed long before the technology emerges. Simply put: if we start thinking about mitigating these problems when they arise, then, most probably, we are too late (van de Poel 2016; Brey 2017).

### Protecting Humans From Evolving Robots

It is hard to overstate the possible implications of the two key enabling features in evolving robots: self-replication and random change in robot form and behavior. First, self-replication allows robots to multiply without human intervention and thus would raise the need for control over their reproduction. Second, mutation or random evolutionary changes in the design of the robots could create undesired robotic behaviors that may harm human interests. Before developing any new technology with such potentially large ramifications, we should determine the acceptability of its consequences and identify ways to anticipate unwanted effects (van de Poel 2016).

Several other fields of science have faced similar safety dilemmas during developments of new technology and subsequent experimentation. In health sciences, biomedical ethical dilemmas are typically evaluated using a principle-based approach, based on the four principles of Beauchamp and Childress (Beauchamp and Childress 2019): autonomy, non-maleficence (avoiding harm), beneficence, and justice. Within the context of technological experimentation, the concept of responsibility has been added (van de Poel 2016), and specifically in the field of Artificial Intelligence (AI), a call has been made for adding the property of “explicability” (Floridi et al., 2018). This property entails that when AI-powered algorithms are used to make morally-sensitive decisions, humans should be able to obtain “a factual, direct, and clear explanation of the decision-making process” (Floridi et al., 2018), or of the decision resulting from the algorithm (Robbins 2019).

In evolutionary robotics all of these principles have clear relevance, but, most pressingly, the risk of harm and the question of responsibility need to be considered in more detail. These, in turn, are intimately related to the crucial issue of control and the potential loss of it. In order for a particular human being or group of human beings to be responsible for some process or outcome, it is usually thought that they need to have some degree of control of the process or outcome. Moreover, loss of control can be viewed as a form of harm, because it is typically seen as undermining human autonomy, and it may compromise other values, such as well-being, which depend to some extent on our ability to control what happens around us.

### Risk of Harm

The issue of risk in the field of AI has previously been considered in relation to control concerns associated with the development of superintelligence (Bostrom 2014; Russell 2019; Russell and Norvig 2020). A notable difference between superintelligence-related concerns and ER-related worries, however, is the perceived probability of the risk. Many people find the idea of superintelligence either inherently implausible or at least something we need not worry about in the short run (Gordon and Nyholm 2021; Müller, 2020). More precisely, people may feel that although an excellent AI chess or Go player is manifestly possible, artificial *general* superintelligence is much less likely to emerge.

In contrast, evolving physical robots need not possess human level intelligence; animal level intelligence in such robots could be sufficient to do significant harm because of their physical features. Even without much individual intelligence and power, the evolved robots could potentially collaborate efficiently and perform much more complex tasks together than they could on their own. In other words, similar to highly social animals such as ants and wasps in the natural world, the number and cooperation among robots could be decisive factors. Therefore, the plausibility of a harmful scenario with evolving robots is all but trivial, and issues of control and the potential loss of it should be considered.

The most difficult aspect in anticipating possible risks of evolving robots is that we would be dealing with an *evolving* system that is inherently and continuously changing. The risk of harm therefore needs to be evaluated for potential future trajectories of the evolutionary process, not only for the current robots. We distinguish three key types of risks associated with the evolutionary process, connected to reproduction, selection, and emergent evolution, respectively:

*Multiplication risk*: The robots can evolve at high reproduction rates, resulting in uncontrolled population growth. If the robot population becomes too large, resources such as space, energy, and raw materials like air or water may be (locally) depleted. This effect can be compared to a locust plague: a swarm’s voracious feeding can completely devour agricultural crops over a vast area, leading to famine and starvation in the human population. While individual robots may not pose any significant risk, their high number and collective behavior can be dangerous.

*Maladaptation risk*: Evolving the robots for a specific task can lead to unwanted features or behaviors that benefit the robot’s assigned task, but that may be harmful to human society. For instance, robots may attempt to dismantle houses to use the stones or cut car tires for the rubber. In the most extreme cases, robots could harm humans if they hinder robots in performing their tasks. This type of risk can evolve because selection is “blind,” meaning the most effective solutions for the task will prevail, without taking other consequences of the evolved trait into account.

*Domination risk*: The robots could evolve to become the dominating “species,” not as a direct effect of selection, but rather as an emergent feature of the robot’s functionality (Badyaev 2011). This can happen if they become superior to humans intellectually, physically, or “emotionally” (being stable and consistent). As a result, they might become benevolent influencers or decision makers, implicitly or explicitly arranging life for us. This effect can be compared to a parent-child relationship where the parent is better in understanding and anticipating situations and therefore confines the spatial range and activities of the child. Even though humans may not be physically harmed by the robot’s dominating behavior, human autonomy would be, at least partly, diminished.

### Meaningful Human Control

The risks of harm associated with robot evolution as identified above all arise from the underlying *control problem* of (semi)autonomous robotic systems. In the Artificial Intelligence literature, solutions to this control problem are often phrased in terms of *meaningful human control* (Santoni de Sio and van den Hoven, 2018). This term acknowledges that whereas there may be no direct control–e.g., a steering wheel in a car–it may still be possible to have *indirect control* allowing for allocation of responsibilities (Di Nucci 2020; Nyholm 2020). For evolving robots this would mean that precautionary design measures are required to control the evolutionary process itself. Such measures could include:

1) *Centralized, externalized reproduction.* A rigorous way of maintaining control over the system would be to set it up such that robot reproduction cannot take place “in the wild” but only in a centralized infrastructure–a reproduction center–where robot offspring can be made, for instance by 3D-printers and automated assembly facilities (Eiben et al., 2013; Hale et al., 2019). Limiting the reproduction to a single or a few centers not only allows keeping track of robot numbers, but also provides the option to restrict the number of robots produced per day. In addition, such a center could provide a possibility to test new robots for safety before releasing them into the outside world. Furthermore, a reproduction center can contain a “kill switch” that can be used to halt evolution by shutting down the reproduction process.
2) *Advanced prediction systems.* Complex simulations and prediction models could provide the necessary previews of the evolutionary process and the emerging features of the resulting robots. Such a “crystal ball,” as Bostrom puts it, would allow humans to anticipate the developments and intervene if necessary (Bostrom 2014). To this end it is important to note that, contrary to natural organisms, robots can be monitored in detail. At the cost of some overhead for inspecting and logging the communications, actions, sensory inputs, and even the internal processes of the robots, a lot of data can be collected and utilized. To be realistic, modeling and predicting the complex evolutionary process of robots in the real world is currently beyond reach. In addition to practical constraints (data collection, data volumes, processing power) there can be fundamental limitations regarding the prediction of emergent behaviors in a population of evolving and interacting robots in environments that are dynamically changing and not fully known. However, meteorological and epidemiological simulations demonstrate that predictions need not be accurate to the finest details to be useful.
3) *Value loading.* Another option for control suggested by Bostrom (Bostrom 2014) is to instill certain properties inside the robot that make sure the robot does not set goals that are risky for humans. For instance, the system might be set up so that robots do not want to reproduce independently, so they will not “revolt” against the centralized reproduction center.

These control measures, meaningful as they are, can leave humans vulnerable because of the very nature of evolving systems, in which change is inherent. Evolving robots represent a whole new breed of machines that can and will change their form and behavior. This implies that robots could adapt their behavior to escape the implemented control measures. Therefore, controlling evolving robots is different from controlling the production of fixed entities, such as cars. One would therefore need to continuously adjust the control measures to stay ahead of evolutionary escape routes, not unlike a co-evolutionary arms race (Thompson 1994). In what follows, we highlight three possible evolutionary escape routes: two technology-related possibilities and one that exploits human emotional vulnerabilities and normative judgments.

First, the robots could develop solutions to circumvent the technological safeguards that have been put into place. A very unlikely, but conceivable escape route is the “Jurassic Park scenario,” where the robots find an alternative way of reproducing outside the central reproduction facility. To mitigate this risk, additional reproductive constraints may be necessary, e.g., using an ingredient that is necessary for being viable and controlling its supply (Ellery and Eiben 2019). A more realistic way of escaping control is that robots stop sharing their operational data and thereby evade monitoring. This could partly be resolved by a mandatory data recorder built into all robots, similar to the flight recorders (a.k.a. black box) in airplanes (Winfield and Jirotka 2017; Winkle 2020).

Second, while Bostrom [36] suggests “value loading” for robotic and AI systems, in the case of evolving robot populations it is important to realize that it would be risky to rely on the (current) features of individual robots. In an evolutionary process the robot’s features undergo change. This does not mean that creating certain features (such as values or goals) in the robots is without merit, but it should be combined with some form of verification that the goals/values continue to be present in the newly produced robots. This requires new technologies that effectively combine immutable values with adaptable robot features and protocols for a thorough screening of “newborn” robots before they are allowed to leave the reproduction facility.

A third possibility for evolving robots to escape human control is non-technological, exploiting deep-seated emotional response patterns. Specifically, humans may grow fond of robots, developing feelings of “affection” towards them (Carpenter 2016; Darling 2017). This emotional vulnerability is probably the result of the long evolutionary history of humans, which has equipped our brains with various motivational and affective pathways tuned to human psychology (Damiano and Dumouchel 2018; Nyholm 2020). Consequently, we are responding to robots with brains and emotional sensitivities that are well-adapted to interacting with fellow human beings and familiar animals, but not necessarily adequate to responding sensibly to machines. Robots and other artificially intelligent technologies, therefore, may “push our Darwinian buttons” in ways that we may not upon reflection find suitable (Turkle 2004).

These sensibilities can be exploited if robots evolve features humans tend to like such as, possibly, big eyes, certain locomotion patterns or “lovely” sounds and gestures. Such features can increase attachment, undermine human controller’s ability to remain objective and provide an evolutionary advantage on the long run. For instance, a robot could entice a human into supplying it with extra energy or allowing it to reproduce. Similarly, a “lovable” robot could prevent a human from switching off the robot or using the “kill switch” to shut down the evolution of the whole robotic species. These scenarios illustrate how emotions could get in the way of strict human control and induce an evolutionary bias [cf. (Bryson 2018)].

### Filling the Responsibility Gap

The above-mentioned considerations concern ways of controlling the process of robot evolution. But there are more conceptual–ethical–concerns as well. Being able to ascribe responsibility is always important when risks are involved, both from an ethical and a legal point of view. The relevant form of responsibility here does not only have a backward-looking component (who can be blamed when things have gone wrong?), but is also forward-looking and clarifies who should do what in order to maintain control, e.g., mitigating risks and taking precautions (Nihlen Fahlquist 2017; Di Nucci 2020). Thus, a prominent issue is a potential responsibility gap. A responsibility gap occurs when there are significant risks of harm for which someone should take responsibility, but there is no obvious candidate to ascribe the responsibility to (Matthias 2004; Sparrow 2007; Nyholm 2020). In the solutions above, the control envisioned will, at least in part, be exercised by humans. The crucial question is then how potential responsibility gaps might be filled.

At this point it may be instructive to refer to recent work by Santoni de Sio and Van den Hoven (Santoni de Sio and van den Hoven, 2018). They have developed a “track-and-trace” account of meaningful human control. The tracking part requires that the system behaves according to rules or paths that track human interests. In other words, the system should behave in a way that aligns with human values and interests. The tracing part requires that the robotic behavior can be traced back to at least one person who understands how the process works, as well as its moral and social significance. It might be added here that, ideally, this should work like when one is tracking and tracing a parcel: it should be possible to monitor how things are developing, just like one can monitor the journey of a parcel [(Nyholm 2020), p. 78].

The track-and-trace theory, understood as including the monitoring condition, looks promising from an ethical perspective for robot evolution. If the robot evolution is tracking human interests, if there are people who understand the process and its moral significance, and are able to monitor the robot evolution, then we can tentatively say that meaningful human control over this process has been achieved. If those conditions are fulfilled, that could help to fill any potential responsibility gaps.

The control solutions suggested above cover the “tracking” requirements from the track-and trace theory to a significant extent. The centralized, externalized reproduction centers would allow humans to monitor the numbers and types of robots produced each day, while the crystal ball would give insight into the future directions of the evolutionary path of the robots. Being able to monitor robot development in these ways, the humans involved would be able to observe whether human interests are being tracked. If not, they could use the “kill switch.” The tracing part however, would need to be developed further as, at the moment, we do not have an appropriate level of understanding nor control of how the evolutionary process unfolds. At the same time, if studying these evolutionary processes in robots would deepen our scientific understanding of evolution, this could in effect help to also fulfil the tracing condition.

That being said, the big challenge here is, again, the inherent variability of an evolutionary system where new features emerge through random mutations and recombination of parental properties. Even though the whole system, specifically the genetic code (the robotic DNA), the mutation operators, and recombination operators are designed by humans, it is not clear to what extent these humans can be held responsible for the effects over several generations. On the positive side, let us reiterate that robots are observable, thus the genetic material and genealogy tree of an evolving population can be logged and inspected. In principle, it is possible to examine a newly created genotype (the robotic zygote) before the corresponding phenotype (the robot offspring) is constructed and destroy the genotype if it fails a safety test.

### Protecting Evolving Robots From Humans

In the sections above, our main concern was to protect the human race from evolving robots. However, the matter can be inverted if we conceive of robots that can evolve and learn as a form of artificial *life*. Considering them as a form of life implies different kinds of ethical considerations (Coeckelbergh 2012; Bryson 2018; Gunkel 2018; Danaher 2020), which go beyond the issues of affection and attachment to individual robots as discussed above, and refer to the whole robotic population. The key is to see the robot population as a species that requires some *moral consideration*. Such an ethical view could be motivated by two arguments.

First, these robots have the possibility of reproduction, and in biology the crucial difference between life and non-life is reproduction. In addition, these robots share other characteristics with other life forms, such as movement and energy consumption. Second, the robots are not only able to reproduce; they themselves have also evolved. In other words, these robots are not (just) the result of human design, but of an evolutionary process. If humans, generally, start to feel that these robots are *forms of life*–albeit artificial–this could entail some perceived moral obligations, like we may feel we have obligations towards whales, dolphins, dogs, and cats. In other words, we may feel that these robots–and along with them, their evolutionary process–deserve some level of protection. This could raise the issue of robot rights, similarly to how we think about animal rights (Gellers 2020).

Second, it could be questioned whether certain control-interventions, such as the use of the “kill switch”, are ethical regarding such forms of artificial life. An essential question here is if terminating evolutionary robots should be seen as switching off a machine or as killing a living being (Darling 2021). In any case, such moral considerations could potentially limit the possibilities of meaningful human control of robot evolution we have discussed.

## Conclusion

Robot evolution is not science fiction anymore. The theory and the algorithms are available and robots are already evolving in computer simulations, safely limited to virtual worlds. In the meanwhile, the technology for real-world implementations is developing rapidly and the first (semi-)autonomously reproducing and evolving robots are likely to arrive within a decade (Hale et al., 2019; Buchanan et al., 2020). Current research in this area is typically curiosity-driven, but will increasingly become more application-oriented as evolving robot systems can be employed in hostile or inaccessible environments, like seafloors, rain-forests, ultra-deep mines or other planets, where they develop themselves “on the job” without the need for direct human oversight.

A key insight of this paper is that the practice of second order engineering, as induced by robot evolution, raises new issues outside the current discourse on AI and robot ethics. Our main message is that awareness must be created before the technology becomes mature and researchers and potential users should discuss how robot evolution can be responsibly controlled. Specifically, robot evolution needs careful ethical and methodological guidelines in order to minimize potential harms and maximize the benefits. Even though the evolutionary process is functionally autonomous without a “steering wheel” it still entails a necessity to assign responsibilities. This is crucial not only with respect to holding someone responsible if things go wrong, but also to make sure that people take responsibility for certain aspects of the process–without people taking responsibility, the process cannot be effectively controlled. Given the potential benefits and harms and the complicated control issues, there is an urgent need to follow up our ideas and further think about responsible robot evolution.

## Footnotes

1 We do not consider evolutionary soft robotics here, because that field mainly focuses on actuators and sensors, not on fully autonomous, untethered (soft) robots.
